# HIV/AIDS-related mortality in Africa and Asia: evidence from INDEPTH health and demographic surveillance system sites

**DOI:** 10.3402/gha.v7.25370

**Published:** 2014-10-29

**Authors:** P. Kim Streatfield, Wasif A. Khan, Abbas Bhuiya, Syed M.A. Hanifi, Nurul Alam, Ourohiré Millogo, Ali Sié, Pascal Zabré, Clementine Rossier, Abdramane B. Soura, Bassirou Bonfoh, Siaka Kone, Eliezer K. Ngoran, Juerg Utzinger, Semaw F. Abera, Yohannes A. Melaku, Berhe Weldearegawi, Pierre Gomez, Momodou Jasseh, Patrick Ansah, Daniel Azongo, Felix Kondayire, Abraham Oduro, Alberta Amu, Margaret Gyapong, Odette Kwarteng, Shashi Kant, Chandrakant S. Pandav, Sanjay K. Rai, Sanjay Juvekar, Veena Muralidharan, Abdul Wahab, Siswanto Wilopo, Evasius Bauni, George Mochamah, Carolyne Ndila, Thomas N. Williams, Sammy Khagayi, Kayla F. Laserson, Amek Nyaguara, Anna M. Van Eijk, Alex Ezeh, Catherine Kyobutungi, Marylene Wamukoya, Menard Chihana, Amelia Crampin, Alison Price, Valérie Delaunay, Aldiouma Diallo, Laetitia Douillot, Cheikh Sokhna, F. Xavier Gómez-Olivé, Paul Mee, Stephen M. Tollman, Kobus Herbst, Joël Mossong, Nguyen T.K. Chuc, Samuelina S. Arthur, Osman A. Sankoh, Peter Byass

**Affiliations:** 1 Matlab HDSS, Bangladesh; 2 International Centre for Diarrhoeal Disease Research, Bangladesh; 3 INDEPTH Network, Accra, Ghana; 4 Bandarban HDSS, Bangladesh; 5 Chakaria HDSS, Bangladesh; 6 Centre for Equity and Health Systems, International Centre for Diarrhoeal Disease Research, Bangladesh; 7 AMK HDSS, Bangladesh; 8 Centre for Population, Urbanisation and Climate Change, International Centre for Diarrhoeal Disease Research, Bangladesh; 9 Nouna HDSS, Burkina Faso; 10 Nouna Health Research Centre, Nouna, Burkina Faso; 11 Ouagadougou HDSS, Burkina Faso; 12 Institut Supérieur des Sciences de la Population, Université de Ouagadougou, Burkina Faso; 13 Institut d’Études Démographique et du parcours de vie, Université de Genève, Geneva, Switzerland; 14 Taabo HDSS, Côte d’Ivoire; 15 Centre Suisse de Recherches Scientifiques en Côte d’Ivoire, Abidjan, Côte d’Ivoire; 16 Université Félix Houphoët-Boigny, Abidjan, Côte d’Ivoire; 17 Swiss Tropical and Public Health Institute, Basel, Switzerland; 18 Kilite-Awlaelo HDSS, Ethiopia; 19 Department of Public Health, College of Health Sciences, Mekelle University, Mekelle, Ethiopia; 20 Farafenni HDSS, The Gambia; 21 Medical Research Council, The Gambia Unit, Fajara, The Gambia; 22 Navrongo HDSS, Ghana; 23 Navrongo Health Research Centre, Navrongo, Ghana; 24 Dodowa HDSS, Ghana; 25 Dodowa Health Research Centre, Dodowa, Ghana; 26 Ballabgarh HDSS, India; 27 All India Institute of Medical Sciences, New Delhi, India; 28 Vadu HDSS, India; 29 Vadu Rural Health Program, KEM Hospital Research Centre, Pune, India; 30 Purworejo HDSS, Indonesia; 31 Department of Public Health, Universitas Gadjah Mada, Yogyakarta, Indonesia; 32 Kilifi HDSS, Kenya; 33 KEMRI-Wellcome Trust Research Programme, Kilifi, Kenya; 34 Department of Medicine, Imperial College, St. Mary’s Hospital, London, United Kingdom; 35 Kisumu HDSS, Kenya; 36 KEMRI/CDC Research and Public Health Collaboration and KEMRI Center for Global Health Research, Kisumu, Kenya; 37 Nairobi HDSS, Kenya; 38 African Population and Health Research Center, Nairobi, Kenya; 39 Karonga HDSS, Malawi; 40 Karonga Prevention Study, Chilumba, Malawi; 41 London School of Hygiene and Tropical Medicine, London, United Kingdom; 42 Niakhar HDSS, Senegal; 43 Institut de Recherche pour le Developpement (IRD), Dakar, Sénégal; 44 Agincourt HDSS, South Africa; 45 MRC/Wits Rural Public Health and Health Transitions Research Unit (Agincourt), School of Public Health, Faculty of Health Sciences, University of the Witwatersrand, Johannesburg, South Africa; 46 Umeå Centre for Global Health Research, Umeå University, Umeå, Sweden; 47 Africa Centre HDSS, South Africa; 48 Africa Centre for Health and Population Studies, University of KwaZulu-Natal, Somkhele, KwaZulu-Natal, South Africa; 49 National Health Laboratory, Surveillance & Epidemiology of Infectious Diseases, Dudelange, Luxembourg; 50 FilaBavi HDSS, Vietnam; 51 Health System Research, Hanoi Medical University, Hanoi, Vietnam; 52 School of Public Health, Faculty of Health Sciences, University of the Witwatersrand, Johannesburg, South Africa; 53 Hanoi Medical University, Hanoi, Vietnam; 54 WHO Collaborating Centre for Verbal Autopsy, Umeå Centre for Global Health Research, Umeå University, Sweden

**Keywords:** HIV/AIDS, tuberculosis, Africa, Asia, Mortality, INDEPTH Network, Verbal Autopsy, InterVA

## Abstract

**Background:**

As the HIV/AIDS pandemic has evolved over recent decades, Africa has been the most affected region, even though a large proportion of HIV/AIDS deaths have not been documented at the individual level. Systematic application of verbal autopsy (VA) methods in defined populations provides an opportunity to assess the mortality burden of the pandemic from individual data.

**Objective:**

To present standardised comparisons of HIV/AIDS-related mortality at sites across Africa and Asia, including closely related causes of death such as pulmonary tuberculosis (PTB) and pneumonia.

**Design:**

Deaths related to HIV/AIDS were extracted from individual demographic and VA data from 22 INDEPTH sites across Africa and Asia. VA data were standardised to WHO 2012 standard causes of death assigned using the InterVA-4 model. Between-site comparisons of mortality rates were standardised using the INDEPTH 2013 standard population.

**Results:**

The dataset covered a total of 10,773 deaths attributed to HIV/AIDS, observed over 12,204,043 person-years. HIV/AIDS-related mortality fractions and mortality rates varied widely across Africa and Asia, with highest burdens in eastern and southern Africa, and lowest burdens in Asia. There was evidence of rapidly declining rates at the sites with the heaviest burdens. HIV/AIDS mortality was also strongly related to PTB mortality. On a country basis, there were strong similarities between HIV/AIDS mortality rates at INDEPTH sites and those derived from modelled estimates.

**Conclusions:**

Measuring HIV/AIDS-related mortality continues to be a challenging issue, all the more so as anti-retroviral treatment programmes alleviate mortality risks. The congruence between these results and other estimates adds plausibility to both approaches. These data, covering some of the highest mortality observed during the pandemic, will be an important baseline for understanding the future decline of HIV/AIDS.

The human immunodeficiency virus (HIV) and the consequent acquired immune deficiency syndrome (AIDS) caused a globally devastating pandemic starting in the late twentieth century. This pandemic caused mortality of such a magnitude as to distort population age–sex distributions in the worst affected areas (1). Now with the advent and roll-out of effective treatment for case management, the situation is improving (2). However, because the pandemic most affected those areas of the world where reliable health data are scarcest, there remain large uncertainties about measuring the impact of HIV/AIDS, with many assessments relying on modelled estimates (3). Thus, in reality, many millions of people attributed with HIV infection and/or AIDS mortality over the course of the pandemic were neither tested for the virus, nor had their deaths certified by physicians.

In the absence of laboratory testing and physician diagnosis, one way of determining the magnitude of HIV/AIDS-related mortality is by using verbal autopsy (VA), involving a structured interview with family or friends after a death (4). The interview material is then used to assign the cause of death. In many settings, particularly at earlier stages, physicians made such assessments of VA data. Recently, it has become more common to use computerised models to attribute cause of death, which are faster, cheaper, and more consistent. Neither approach can be regarded as absolutely correct. Following a VA interview, assigning a death as due to HIV/AIDS is not entirely straightforward, because HIV-infected people may die of a variety of causes. As well as the wasting syndromes typical of AIDS deaths, other causes of death, including particularly pulmonary tuberculosis (PTB) and pneumonia, occur at higher rates and in different age groups among HIV-infected people.

In this paper, we present HIV/AIDS-specific mortality rates as determined by computer-interpreted VA from 22 INDEPTH Network Health and Demographic Surveillance Sites (HDSS) across Africa and Asia (5). These findings are complemented with the corresponding rates for PTB and pneumonia. Although these HDSSs are not designed to form a representative network, each one follows a geographically defined population longitudinally, systematically recording all death events and undertaking verbal autopsies on all deaths that occur. Our aim is to present the HIV/AIDS mortality patterns at each site, comparing these community-level findings with other estimated information on HIV/AIDS in Africa and Asia.

## Methods

The overall INDEPTH dataset (6) from which these HIV/AIDS-specific analyses are drawn is described in detail elsewhere (7). The methods used are summarised in Box 1. Briefly, it documents 111,910 deaths in 12,204,043 person-years of observation across 22 sites. The Karonga site in Malawi did not contribute VAs for children.

*Box 1*. Summary of methodology based on the detailed description in the introductory paper (7)

**Age–sex–time standardisation**

To avoid effects of differences and changes in age–sex structures of populations, mortality fractions and rates have been adjusted using the INDEPTH 2013 population standard (8). A weighting factor was calculated for each site, age group, sex, and year category in relation to the standard for the corresponding age group and sex, and incorporated into the overall dataset. This is referred to in this paper as age–sex–time standardisation in the contexts where it is used.

**Cause of death assignment**

The InterVA-4 (version 4.02) probabilistic model was used for all the cause of death assignments in the overall dataset (9). InterVA-4 is fully compliant with the WHO 2012 Verbal Autopsy standard and generates causes of death categorised by ICD-10 groups (10). The data reported here were collected before the WHO 2012 VA standard was available, but were transformed into the WHO 2012 and InterVA-4 format to optimise cross-site standardisation in cause of death attribution. For a small proportion of deaths, VA interviews were not successfully completed; a few others contained inadequate information to arrive at a cause of death. InterVA-4 assigns causes of death (maximum 3) with associated likelihoods; thus cases for which likely causes did not total to 100% were also assigned a residual indeterminate component. This served as a means of encapsulating uncertainty in cause of death at the individual level within the overall dataset, as well as accounting for 100% of every death.

**Overall dataset**

The overall public-domain dataset (6) thus contains between one and four records for each death, with the sum of likelihoods for each individual being unity. Each record includes a specific cause of death, its likelihood and its age–sex–time weighting.

The InterVA-4 ‘high’ HIV/AIDS setting was used for sites in Kenya, Malawi, and South Africa. All other sites used the ‘low’ setting; the ‘very low’ setting was not used. The InterVA-4 guideline is that the ‘high’ setting is appropriate for an expected HIV/AIDS cause-specific mortality fraction (CSMF) higher than about 1%, though it does not result in any great dichotomisation of outputs; the clinical equivalent is a physician’s knowledge that his/her current case comes from a setting where HIV/AIDS is more or less likely, irrespective of that current case’s particular symptoms. The validity of the InterVA-4 model in assigning HIV/AIDS as a cause of death in relation to HIV sero-status has been extensively explored in conjunction with the ALPHA Network (11), and found to be over 90% specific. Sensitivity is more difficult to assess, since not all people infected with HIV evidently die of AIDS. The same validation exercise pointed to large numbers of cases of PTB and pneumonia as causes of death among the HIV-positive.

Deaths assigned to HIV/AIDS, and the closely related causes of PTB and pneumonia, were extracted from the overall data set together with data on person-time exposed by site, year, age, and sex. As each HDSS covers a total population, rather than a sample, uncertainty intervals are not shown.

For the sake of comparison with other estimates of HIV/AIDS-related mortality, unadjusted data were extracted for all sites for the period 2008–2012 (excluding data from the Farafenni, The Gambia; Purworejo, Indonesia; and FilaBavi, Vietnam sites which did not report for that period). These data were grouped into three age bands (0–14, 15–49, and 50 +) and aggregated by country, to facilitate comparison with contemporaneous national point estimates for 2010.

In this context, all of these data are secondary datasets derived from primary data collected separately by each participating site. In all cases, the primary data collection was covered by site-level ethical approvals relating to on-going health and demographic surveillance in those specific locations. No individual identity or household location data were included in the secondary data and no specific ethical approvals were required for these pooled analyses.

## Results

In the overall dataset, there were 10,455.4 deaths attributed to HIV/AIDS (including fractions of 11,972 individual deaths), with a further 10,363.4 deaths attributed to acute respiratory infections (including pneumonia), and 12,874.8 attributed to PTB.

**Fig. 1:**
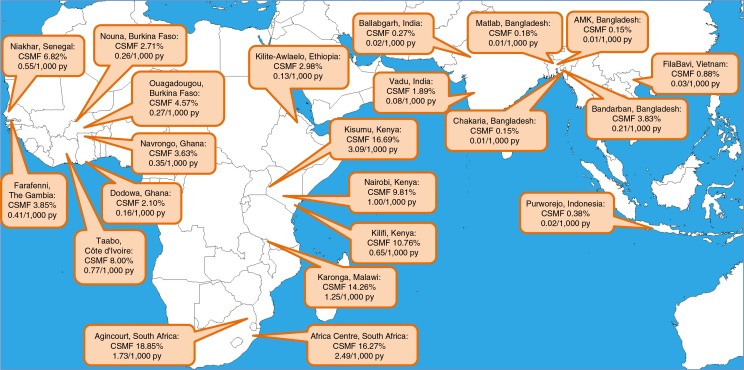
Map showing participating sites, with age–sex–time adjusted cause-specific mortality fractions and adjusted mortality rates for HIV/AIDS.

The age–sex–time standardised CSMFs for HIV/AIDS at each site are shown, together with the population-based HIV/AIDS-specific mortality rate per 1,000 person-years, in Fig. 1. In West African sites, HIV/AIDS CSMF ranged from 2.10 to 8.00%, with HIV/AIDS-specific adjusted mortality rates ranging from 0.16 to 0.77 per 1,000 person-years. In eastern and southern Africa, except Ethiopia, CSMFs were 9.81–18.85%, with rates from 0.65 to 3.09 per 1,000 person-years. In Asia, CSMFs were 0.15–3.83%, with rates from 0.01 to 0.21 per 1,000 person-years.

**Fig. 2:**
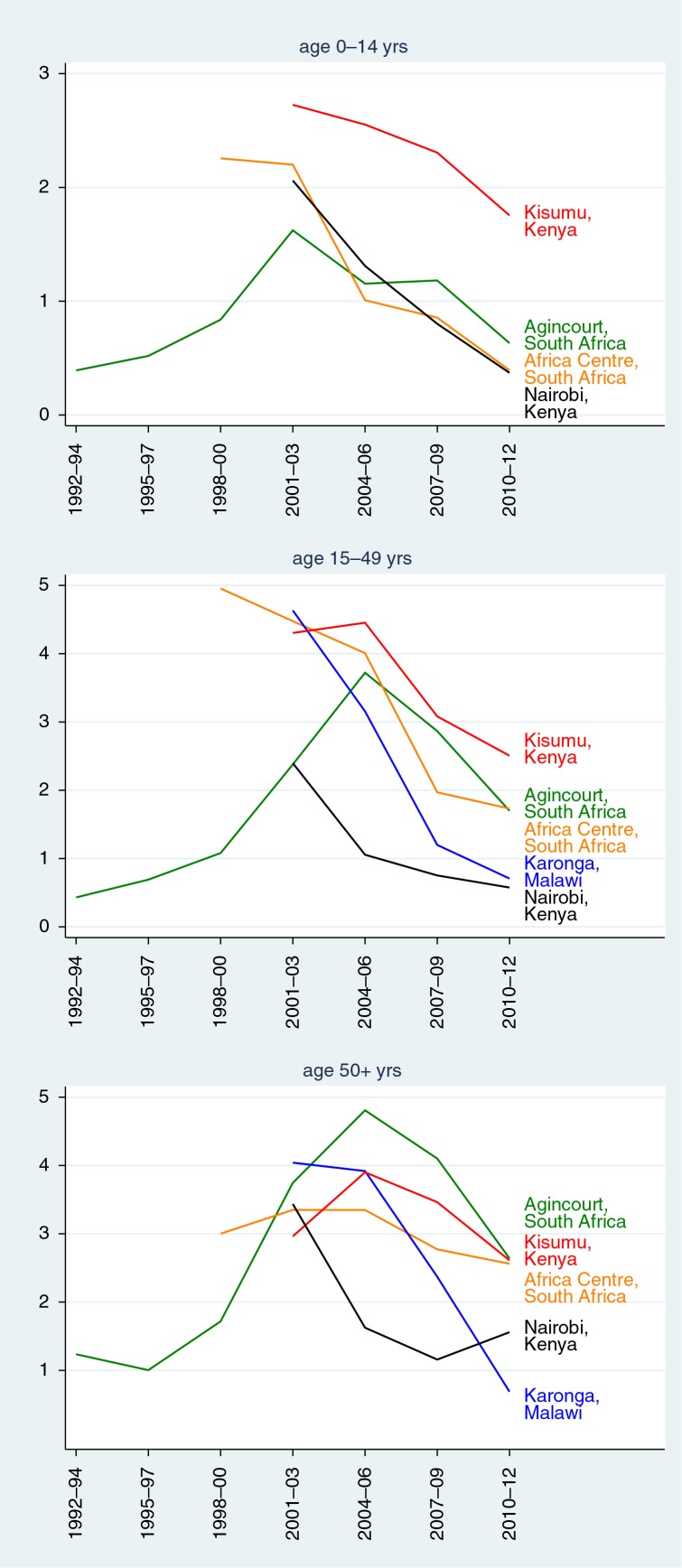
Epidemic curves for HIV/AIDS mortality rates for the five sites where overall HIV/AIDS mortality exceeded 1 per 1,000 person-years.

Figure 2 shows HIV/AIDS mortality epidemic curves for the five sites where overall HIV/AIDS mortality was at least 1 per 1,000 person-years. Apart from the Agincourt, South Africa, site, for which a more or less complete epidemic curve can be seen, the other sites recorded mortality during a period of mainly declining HIV/AIDS mortality.

Table 1 gives HIV/AIDS-specific mortality rates by age group and site. During infancy, the highest HIV/AIDS-specific mortality rate was reported from the Africa Centre, South Africa (7.00 per 1,000 person-years), contrasting with a zero rate from several Asian sites. For the 1–4 age group, the Kisumu, Kenya, site recorded the highest rate (5.40 per 1,000 person-years). In the 5–14 year age group, Asian sites recorded rates from 0 to 0.07 per 1,000 person-years, compared with African sites from 0.02 to 0.40 per 1,000 person-years. In adulthood, the ranges across Asian sites for 15–49 years, 50–64 years, and 65+ years were 0–0.23, 0.02–0.66, and 0–0.09, respectively. Similarly for African sites, ranges were 0.08–3.65, 0.37–4.56, and 0–2.26, respectively.

**Table 1:** HIV/AIDS-specific deaths and mortality rates per 1,000 person-years, by age group and site, from 111,910 deaths in 12,204,043 person-years of observation across 22 sites

	Age group at death					
	
Country: Site	Infant	1–4 years	5–14 years	15–49 years	50–64 years	65+ years
Bangladesh: Matlab						
Adjusted deaths	1.71	7.23	2.18	4.16	3.17	0.00
Rate/1,000 py	0.04	0.04	0.01	0.00	0.02	0.00
Bangladesh: Bandarban						
Adjusted deaths	1.19	0.00	1.00	7.02	3.89	0.00
Rate/1,000 py	0.96	0.00	0.07	0.23	0.66	0.00
Bangladesh: Chakaria						
Adjusted deaths	0.36	0.00	0.00	0.00	1.71	0.00
Rate/1,000 py	0.06	0.00	0.00	0.00	0.11	0.00
Bangladesh: AMK						
Adjusted deaths	0.00	1.44	0.87	0.74	1.00	0.00
Rate/1,000 py	0.00	0.03	0.01	0.00	0.02	0.00
Burkina Faso: Nouna						
Adjusted deaths	7.92	51.12	8.89	88.29	20.34	3.11
Rate/1,000 py	0.26	0.49	0.05	0.32	0.43	0.11
Burkina Faso: Ouagadougou						
Adjusted deaths	11.55	16.15	4.63	19.85	7.57	0.00
Rate/1,000 py	1.66	0.58	0.09	0.17	0.66	0.00
Côte d’Ivoire: Taabo						
Adjusted deaths	10.20	20.09	8.24	28.95	7.23	5.06
Rate/1,000 py	2.57	1.55	0.27	0.60	1.04	1.59
Ethiopia: Kilite Awlaelo						
Adjusted deaths	1.91	2.91	0.87	4.85	4.13	1.89
Rate/1,000 py	0.60	0.22	0.02	0.08	0.37	0.27
The Gambia: Farafenni						
Adjusted deaths	13.14	44.29	11.86	40.20	20.01	3.86
Rate/1,000 py	1.15	1.03	0.13	0.29	0.89	0.34
Ghana: Navrongo						
Adjusted deaths	31.16	92.71	24.70	195.22	52.08	10.61
Rate/1,000 py	1.03	0.80	0.08	0.37	0.41	0.15
Ghana: Dodowa						
Adjusted deaths	5.09	10.80	10.01	41.98	13.51	2.86
Rate/1,000 py	0.36	0.19	0.07	0.16	0.37	0.11
India: Ballabgarh						
Adjusted deaths	0.00	1.44	0.61	2.66	2.21	0.00
Rate/1,000 py	0.00	0.05	0.01	0.01	0.07	0.00
India: Vadu						
Adjusted deaths	8.42	0.00	0.00	2.25	1.23	0.00
Rate/1,000 py	1.96	0.00	0.00	0.02	0.08	0.00
Indonesia: Purworejo						
Adjusted deaths	0.00	0.00	1.34	0.00	3.35	1.98
Rate/1,000 py	0.00	0.00	0.03	0.00	0.12	0.09
Kenya: Kilifi						
Adjusted deaths	69.17	70.79	60.88	276.26	99.83	50.99
Rate/1,000 py	1.80	0.48	0.20	0.65	1.52	1.54
Kenya: Kisumu						
Adjusted deaths	297.87	780.55	128.47	1708.84	406.33	132.85
Rate/1,000 py	7.47	5.40	0.40	3.65	4.56	1.98
Kenya: Nairobi						
Adjusted deaths	66.99	81.34	18.42	356.93	44.46	4.34
Rate/1,000 py	4.67	1.30	0.17	0.93	1.79	0.77
Malawi: Karonga						
Adjusted deaths	n/a	n/a	n/a	226.10	50.15	16.82
Rate/1,000 py				1.92	3.39	1.48
Senegal: Niakhar						
Adjusted deaths	2.68	16.06	11.39	62.15	21.95	7.05
Rate/1,000 py	0.30	0.50	0.19	0.64	1.37	0.66
South Africa: Agincourt						
Adjusted deaths	140.09	302.84	67.11	1434.89	309.61	142.86
Rate/1,000 py	3.81	2.03	0.18	1.98	3.35	2.26
South Africa: Africa Centre						
Adjusted deaths	157.25	232.14	55.38	1227.58	210.72	74.44
Rate/1,000 py	7.00	2.54	0.24	3.28	3.84	1.90
Vietnam: FilaBavi						
Adjusted deaths	0.32	0.00	0.00	3.02	2.80	0.00
Rate/1,000 py	0.15	0.00	0.00	0.04	0.17	0.00

**Fig. 3:**
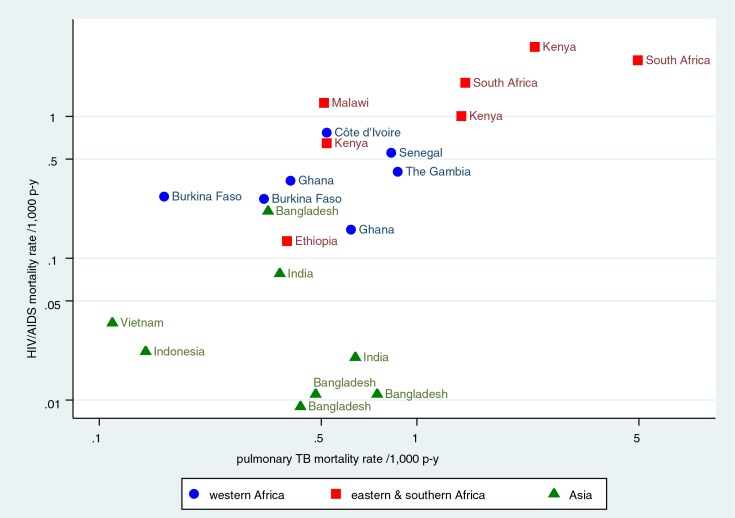
Relationship between HIV/AIDS and pulmonary TB age–sex–time standardised mortality rates for 22 INDEPTH Network sites in Africa and Asia.

Figure 3 shows the relationships between age–sex–time standardised HIV/AIDS mortality rates and PTB mortality rates for all 22 sites. Seven of the eight sites in Asia had an HIV/AIDS rate below 0.1 per 1,000 person-years, but PTB rates ranged from 0.11 to 0.75 per 1,000 person-years. Conversely, six of the seven sites in eastern and southern Africa had HIV/AIDS rates above 0.5 per 1,000 person-years, with PTB rates ranging from 0.52 to 4.96 per 1,000 person-years. The highest age–sex–time standardised HIV/AIDS mortality rate ratio was between Kisumu, Kenya, and AMK, Bangladesh, at 343:1.

**Fig. 4:**
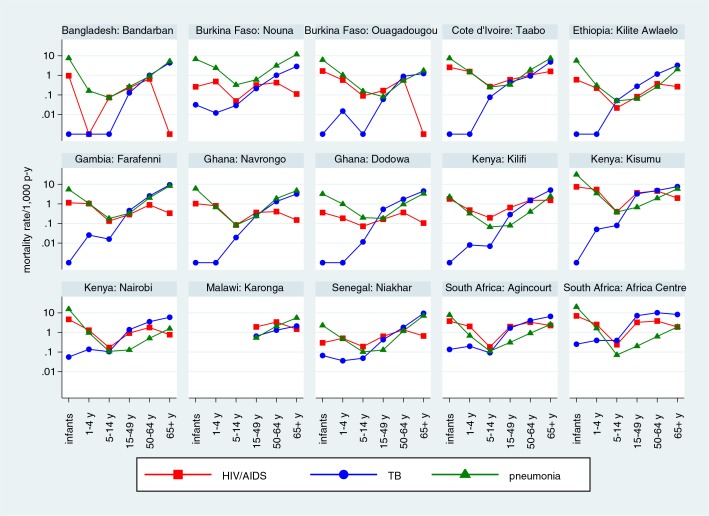
Mortality rates for HIV/AIDS, pulmonary TB, and pneumonia, by site and age group at 15 INDEPTH Network sites for which the overall rate of HIV/AIDS mortality exceeded 0.1/1,000 person-years.

Figure 4 shows HIV/AIDS mortality rates for 15 sites which had an overall HIV/AIDS-specific mortality rate over 0.1 per 1,000 person-years, by age group, also showing corresponding data for PTB and pneumonia. Logarithmic scales have been used to visualise both high and low levels of mortality while using the same scale for each site.

Table 2 shows the INDEPTH results in comparison with national estimates from the UNAIDS Spectrum model (12) and Global Burden of Disease 2010 (13). Longitudinal INDEPTH data were aggregated over 2008–2012 (for the 19 sites reporting for that period) for the purposes of comparison with the Spectrum and Global Burden of Disease 2010 (GBD 2010) estimates, together with corresponding estimates for PTB.

**Table 2:** Within-country estimates of cause-specific (per 1,000 population) mortality rates for HIV/AIDS and TB for 2010 according to UNAIDS Spectrum and the Global Burden of Disease 2010, compared to the equivalent rates across 19 INDEPTH sites, aggregated within 10 countries, for 2008–2012 (per 1,000 person-years)

	HIV/AIDS mortality rates per 1,000														
	
	0–14 years			15–49 years			50+ years			All ages			TB mortality rates per 1,000		
	
Country	Spectrum	GBD 2010	INDEPTH	Spectrum	GBD 2010	INDEPTH	Spectrum	GBD 2010	INDEPTH	Spectrum	GBD 2010	INDEPTH	Spectrum	GBD 2010	INDEPTH
Bangladesh	0.0004	0.0003	0.016	0.004	0.002	0.015	0.002	0.001	0.034	0.002	0.002	0.019	0.533	0.180	0.436
Burkina Faso	0.207	0.273	0.234	0.521	0.588	0.132	0.455	0.369	0.276	0.371	0.429	0.188	0.124	0.216	0.133
Côte d’Ivoire	0.477	0.509	0.804	2.077	2.021	0.597	1.802	1.022	1.212	1.379	1.286	0.749	n/a	0.309	0.430
Ethiopia	0.417	0.185	0.101	1.114	0.472	0.082	0.827	0.160	0.329	0.776	0.320	0.124	0.519	0.453	0.408
Ghana	0.227	0.293	0.212	0.913	1.116	0.217	0.737	0.569	0.329	0.626	0.735	0.232	0.141	0.157	0.479
India	0.034	0.030	0.061	0.179	0.267	0.015	0.083	0.119	0.051	0.119	0.171	0.033	0.299	0.346	0.538
Kenya	0.744	0.617	0.846	2.446	2.201	1.112	1.879	1.371	2.001	1.671	1.452	1.082	0.304	0.257	0.853
Malawi	1.584	1.694	n/a	4.910	5.656	0.854	3.763	3.343	1.140	3.279	3.614	n/a	0.469	0.431	0.268
Senegal	0.082	0.076	0.178	0.184	0.295	0.483	0.075	0.279	1.564	0.129	0.198	0.474	0.228	0.210	0.861
South Africa	1.449	1.166	0.695	10.628	8.048	1.935	9.031	2.422	2.917	7.641	5.099	1.587	1.970	0.322	2.483

## Discussion

Against the background of extensive modelling approaches that have been applied to HIV/AIDS mortality, this dataset presents results from individually documented deaths at a range of sites across Africa and Asia. The expected huge differences in HIV/AIDS mortality rates between Africa and Asia were evident from these results, and, to a lesser extent, the substantial differences that occurred within the African continent. The good news is that HIV/AIDS deaths declined in recent years in all the sites with high mortality rates (Fig. 2), as the effects of prevention and treatment programmes took effect. The interpretation of findings at individual sites depends on local characteristics (14–35) . Two sites, Ouagadougou in Burkina Faso and Nairobi in Kenya, followed urban populations. Bandarban in Bangladesh is located in a militarised frontier zone close to the Myanmar border, which may be associated with higher rates of HIV/AIDS mortality compared with other sites in Bangladesh.

The validity of VA cause of death assignment for HIV/AIDS is not straightforward. In these results, the similar and marked changes over time in the high mortality sites (Fig. 2) added veracity to the InterVA-4 outputs, since the model had no information about the progress of the epidemic over time. Similarly, the extremely low levels of HIV/AIDS-related death assigned as a cause in countries such as Bangladesh and India confirmed the specificity of the methods used. A previous assessment of InterVA-4 validity versus HIV sero-status showed high specificity, but sensitivity was unmeasurable since not all HIV-positive people go on to die from HIV/AIDS (11). However, the same study also showed high mortality rate ratios for PTB and pneumonia between HIV positive and negative cases. ICD-10 classification (36) suggests that almost all HIV-related deaths should be classified under the B20-B24 rubrics, but this is easier said than done in practice, either when using VA or when certifying a death, if there is no evidence of HIV status. In view of the apparently complex relationships between HIV/AIDS deaths and PTB deaths in different settings, as evidenced in Fig. 3, it is not simply a matter of adding together HIV/AIDS and PTB deaths across all settings. However, the total of what InterVA-4 assigns as HIV/AIDS and PTB deaths may provide a better approximation of the overall burden of HIV/AIDS-related mortality for at least the 15–49 year age group in high HIV settings. The question of HIV/AIDS-related mortality associated with pregnancy has also been a matter of debate (37). Another paper in this series analyses pregnancy-related mortality in detail, including the attribution of HIV/AIDS-related deaths between indirect maternal and incidental categories (38).

The WHO 2012 VA standard (39) includes an indicator relating to previous diagnosis of HIV, although the validation study suggested that this was seriously under-reported in VA interviews (11). The WHO 2012 standard, and therefore InterVA-4, does not yet include any details of anti-retroviral therapy (ART), although that will become a more pressing issue as experience of mortality patterns among HIV positive individuals with long exposure to ART develops. It is as yet a relatively open question as to what the major causes of death among HIV-positive people might be after possible decades of ART.

There are other major pieces of work describing HIV/AIDS mortality patterns across Africa and Asia, but these largely relied on modelling estimates from whatever specific sources of data were available, and therefore carried large degrees of uncertainty given the sparse nature of the data from many settings. The two major sources of contemporaneous estimates for HIV/AIDS mortality come from the UNAIDS Spectrum model (12) and the GBD 2010 model (13). Although our purpose here is not to compare these two models with each other, it is worth noting that there are some major differences. For example, among the countries represented here, the estimates for Ethiopia vary three-fold.

Table 2 shows estimates of HIV/AIDS-related and PTB mortality rates for 12 countries according to Spectrum, GBD 2010, and InterVA-4, which in many cases were very similar, though with differences in places. It must be remembered that these comparisons were compromised by taking INDEPTH sites that are not designed to be nationally representative and putting their findings alongside modelled estimates that are intended to reflect national situations. In South Africa, it appeared that InterVA-4 assigned a substantial amount of HIV/AIDS mortality as PTB, which is perhaps unsurprising in that high-prevalence setting. InterVA-4 arrived at a substantially higher HIV/AIDS mortality estimate than Spectrum for Senegal, and vice-versa for India. There were also many similarities in PTB mortality rates, though differences were evident in Ghana, Kenya, and Senegal.

Similarly there were relatively few appreciable differences between GBD 2010 and InterVA-4 estimates. The differences may reflect local disparities in rates between sites and national populations, given that the relationships between symptoms and causes would not be expected to vary substantially between countries. It also has to be remembered that, although all these VAs have been processed in a standardised way using the WHO 2012 protocol, they were collected in the field in slightly different ways before 2012, and some observed differences may also reflect that. Overall, however, there was appreciable congruence in mortality rates between these various sources.

## Conclusions

Measuring HIV/AIDS mortality continues to be a highly challenging area, particularly in Africa, where rates are high and data are often unavailable. This is the largest single systematic study that has applied common methodologies to HIV/AIDS mortality at the individual level across Africa and Asia, and it largely confirms the corresponding findings coming from modelled estimates. This mutually adds plausibility to both existing estimates and to these population-based findings. The challenges involved in measuring HIV/AIDS mortality will grow as ART coverage and individual duration on treatment increase; in many ways, these results represent an important baseline for future studies of the treated pandemic.
